# Discoloration of Roots Caused by Residual Endodontic Intracanal Medicaments

**DOI:** 10.1155/2014/404676

**Published:** 2014-02-09

**Authors:** Belinda Kuan-Jung Chen, Roy George, Laurence James Walsh

**Affiliations:** ^1^School of Dentistry and Oral Health, Griffith University, Gold Coast Campus, Southport 4215, Australia; ^2^The School of Dentistry, University of Queensland, Brisbane, QLD 4000, Australia

## Abstract

*Aims*. This study examined the extent to which intervisit corticosteroid-based antibiotic pastes (CAP) medicaments contribute to staining of tooth structure after attempted removal by irrigation techniques. *Methods*. A total of 140 roots were prepared and the canals were filled with Ledermix paste (demeclocycline), Odontopaste (clindamycin), and Doxypaste (doxycycline). The pastes were removed after 2 or 4 weeks of storage in the dark using EDTA and NaOCl with either a 27-gauge-slotted needle or an EndoActivator (Dentsply). The roots were then exposed to an intense light source for 30 minutes each week and photographed after a further 1, 3, or 6 months. Digital images were standardized and data for changes in luminosity were analysed using repeated measures ANOVA and a post hoc test. *Results*. Removal of the medicament did not prevent later discolouration. There was no significant difference between the paste removal methods. Ledermix paste caused the greatest darkening compared to the untreated controls, for both application periods and both methods of removal. Doxypaste and Odontopaste caused less darkening than Ledermix. *Conclusion*. Medicaments that stain teeth may continue to discolour teeth despite best attempts to remove them. This study stresses the importance of material selection and minimising contact of Ledermix within the coronal aspects of teeth.

## 1. Introduction

Corticosteroid-based antibiotic-containing pastes (CAP) are used as short- and medium-term intracanal medicaments because they exert both anti-inflammatory and antibiotic actions [1–11], which are useful in managing periapical inflammation and root resorption [1, 2, 10, 12–18]. Diffusion of triamcinolone and demeclocycline from the CAP product Ledermix (Lederle Pharmaceuticals, Wolfratshausen, Germany), which contains 1% triamcinolone acetonide and 3.21% demeclocycline HCl, through radicular dentine occurs readily, reaching a peak after only two hours [19]. The rate of release of demeclocycline falls to less than one-tenth of the initial release rate by the 7th day [1, 3, 6, 19, 20].

Binding of demeclocycline from CAP to dentine and its subsequent photooxidation when exposed to light can cause intense staining [7, 19, 21]. To address this potential problem, other CAP have been developed, including Odontopaste (Australian Dental Manufacturing, Brisbane, Australia) with 1% triamcinolone acetonide and 5% clindamycin HCl and Doxypaste (Ozdent, Castle Hill, Australia) with 1% triamcinolone acetonide and 3% doxycycline hyclate. All three pastes contain the same underlying vehicle of polyethylene glycol to which various excipients and fillers are added, which do not exert antimicrobial activities. Compared with demeclocycline, doxycycline is more active as an antibiotic [22, 23] and poses less risk of staining [24]. Past laboratory studies of root discolouration from use of Ledermix CAP demonstrated an effect of ambient sunlight [9, 10], but the irradiation parameters could not be controlled rigidly in terms of irradiance and fluence. Clinical studies likewise demonstrate discolouration of replanted avulsed teeth over 12 months from Ledermix CAP placed into the root for 60–90 days [15].

Recommendations for use of CAP range from two weeks to two months [25]. It is unknown whether existing methods of removing CAP, such as flushing the root canal system using various irrigants using conventional open-ended needles, are effective in removing all the material, thereby preventing subsequent staining of roots. The EndoActivator (Dentsply Maillefer, Ballaigues, Switzerland) is a sonic agitation device which according to supplier has been designed “to safely and vigorously energize intracanal irrigants during endodontic treatment.” The device employs plastic tips to agitate irrigant solutions at between 2,000 and 10,000 cycles per second to enhance their cleaning actions [26, 27]. This approach using mechanical agitation may be preferable to irrigation from a syringe in terms of removing remnants of paste from the canal.

This study was undertaken to evaluate and track the development of root discolouration, after an attempted removal of the intervisit corticosteroid-based antibiotic pastes (CAP) medicaments using either a conventional irrigation technique or an EndoActivator.

## 2. Materials and Methods

Extracted single rooted human teeth with finished root development and single patent canals and closed apices (*n* = 140) were collected with approval of the institutional ethics committee. The teeth were free of discolouration or translucency, fractures, restorations or dental caries affecting the root surface, or external resorption. After being placed in 1% NaOCl for 20 minutes to degrade external soft tissue remnants, the teeth were rinsed in water and the external root surface debrided using an ultrasonic scaler. The majority of the crown was removed from each tooth using a diamond disc to give roots of equal length (12 mm). The outer root surfaces were treated using aluminium oxide abrasive discs from coarse to fine grit to give a standardized matt finish. Roots were then stored in 0.2% thymol solution until used [28]. Working length of teeth was established by passing a size #8 K file past the apical foramen and backing off by 1 mm. Canals were then prepared to working length using F3 size nickel-titanium rotary ProTaper files (Dentsply International), under constant irrigation with alternating 1% NaOCl (Milton) and 15% EDTA/C (Endosure, Dentalife, Croydon, Australia) solutions. A final rinse sequence involving EDTA/C for 2 minutes was performed to ensure complete removal of smear layer. The canals were dried with paper points, and the roots stored in sealed containers for 7 days in ambient room light. The rationale for storage under ambient light was to reduce the level of available chlorine from any remnants of NaOCl without introducing additional reagents [29]. The roots remained moist during this initial one-week period of storage. After recording baseline images, roots were allocated randomly into 14 subgroups of 10 each (Table 1), and the canals filled completely with the respective CAP (Ledermix, DoxyPaste, Odontopaste) using a 5 mL syringe with a small tip placed apically as far as possible under subdued lighting conditions. Injection of the pastes was continued until paste was seen to extrude from the apical foramen, and then the canal backfilled to the level of the cut surface. Any excess paste on the cut root surface and at the apical foramen was removed immediately using an alcohol wipe. No paste material was allowed to contact the lateral aspects of the roots. The prepared roots containing CAP were then kept in complete darkness at 37°C and 100% humidity for either 2 or 4 weeks. Roots without CAP were run in parallel as negative controls.

## 3. Removal of Medicaments

Canals were rinsed for one minute each using EDTA and then NaOCl. These irrigants were delivered in one of two ways. The timing of the interventions was based on manufacturer instructions for the EndoActivator (Dentsply Maillefer, Ballaigues, Switzerland) which recommend agitation of fluids for 30–60 seconds. In both groups, the bulk of the paste was first removed using a hand held ProTaper F3 file introduced in the canal briefly and rotated one-half turn to scrape the walls lightly. EDTA was then placed into the canal with a 27-gauge Monoject open-ended notched needle tip (Kendall Healthcare, Mansfield, QLD). In the conventional irrigation group, the needle tip was placed 1 mm short of the working length, and the canal flushed for one minute with EDA during which time the needle tip was moved manually to obtain gentle manual agitation and thus more effectively flush material from the canal. The EDTA was then flushed out with a syringe and replaced with NaOCl, which was then flushed through the canal for a further minute.

In the second method, after the bulk of the paste had been removed using the hand held ProTaper file, EDTA was placed into the canal with a syringe, and the solution agitated for one minute using an EndoActivator (Dentsply Maillefer) fitted with an ISO 25 size disposable plastic tip. The EDTA was then flushed out with a syringe and replaced with NaOCl, which was agitated with the EndoActivator for a further minute, using the same tip. After removal of CAP, all canals were then dried with paper points before proceeding to the light exposure phase of the study.

## 4. Light Exposure

Roots were subjected to irradiation for 30 minutes once per week over the following 6 months using a 35 Watt Xenon HID spotlight which served as an artificial sunlight source replicating ground level sunlight (colour temperature 6000 Kelvin) [30]. This light was used at a 40 cm target distance, giving an intensity of 29.4 million mean spherical candelas and a flux of 3300 lumens. The spectral distribution of the light source was analysed with a spectrometer (Model USB2000, Ocean Optics, Dunedin, FL, USA). Samples were stored in the dark at 37°C and 100% humidity between light exposure and photographed after 1, 3, and 6 months. Roots which had been prepared but had not been treated with CAP were run in parallel to control a possible effect of either light or storage alone [31] on the colour of roots.

## 5. Data Collection and Analysis

Roots were photographed using a Canon EOS 500D SLR camera fitted an EF 100 mm f/2.8 Macro USM lens and an MR-14EX ring flash was used (Canon Inc., Tokyo, Japan) under constant fixed exposure settings. The camera was mounted on a tripod at a fixed distance of 23 cm away from the samples. Each root was fixed into a specially modified immunoassay microtiter plate, allowing standardized views of both sides of the root in longitudinal view. The baseline image data for each sample therefore provides the relevant reference point, from which subsequent changes occur. Each photographic image included a colour reference beside the sample as an internal quality control for image exposure.

Image analysis was undertaken using Adobe Photoshop CS3 software. All images of tooth were first traced using the Magic Lasso tool and then the histogram tool showed the mean luminosity values of the entire root. This value was then adjusted to the luminosity values of each colour reference. All luminosity values were on a scale of 256 points, with zero indicating black and 256 representing white. Because each tooth started from a different colour and the individual baseline served as the reference point against which later changes would be compared, the analysis was based on a repeated measures assessment of changes in luminosity (darkness) for each root. Data sets were analysed using the repeated measure ANOVA (Friedman's test) and a post hoc Dunn's tests.

## 6. Results

**Figure 1:**
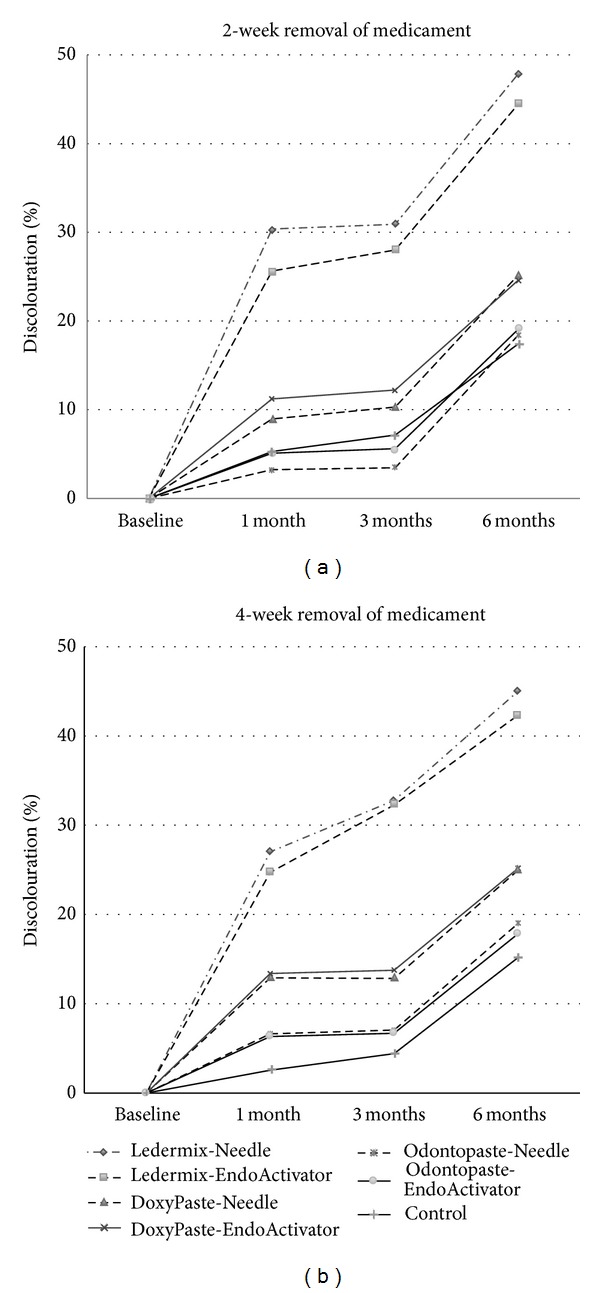
Effect of paste type, paste removal method, and time on normalized luminosity of roots. Graphs show change in a percentage of discolouration over time. (a) shows data for paste exposure of 2 weeks and (b) for 4 weeks. Each data series shows the mean of 10 roots and each data point on the timeline represents the mean of 20 images at that storage time (2 per root to show both sides). Error bars have been omitted for clarity.

The results showed the independent effect of several variables as being statistically significant: type of CAP, exposure time to CAP, and the storage time after paste removal. There was no significant effect for the variable of paste removal method (Tables 2 and 3). To allow better visualisation of the effects of changes in luminosity over the time, data in Figures 1(a) and 1(b) was described in percentage as a proportion of change over time from baseline (week 0) to 6 months. The mean reduction in luminosity in control roots, which was due to light and storage alone, was 16% at 6 months.

**Figure 2:**
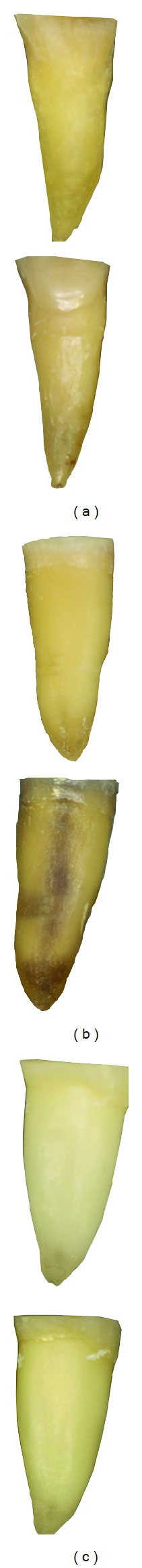
Effect of intense light on roots following removal of root canal medicaments using EndoActivator. (a) DoxyPaste; (b) Ledermix paste; (c) Odontopaste. Upper row, baseline image of root canal with medicaments and no light exposure at the end of 2 weeks. Bottom row, images at 6 months of root canals following removal of medicament at 2 weeks and intense light exposure (30 min/week).

In roots containing CAP, the mean luminosity declined progressively from baseline after removal of the paste, causing a corresponding increase in the percentage change in luminosity (percentage discolouration), as shown in Figure 1. The reduction from baseline in both the conventional irrigation and CAP groups was significant, but there was no difference between the two paste removal protocols at any time point. The progressive darkening was most dramatic in the roots with Ledermix CAP, regardless of the method of paste removal. Less severe progression of discolouration over time was seen with Doxypaste and the least change of all occurred with Odontopaste (Figures 1 and 2; Tables 2 and 3). The discolouration was significantly greater for Ledermix compared to both Doxypaste and Odontopaste groups at 2, 3, and 6 months for both the 2-week and 4-week treatment times and for both paste removal techniques. While there was a significant difference between DoxyPaste and Odontopaste for roots treated for only 2 weeks, there was no difference between these when roots were treated for 4 weeks.

Changes in luminosity at 2 weeks and 4 weeks were different between groups (*P* < 0.0001), with the Ledermix groups showing the greatest discolouration compared to the untreated control (*P* < 0.001) and to other treatment groups (*P* < 0.01 or *P* < 0.05). In each series of CAP groups, there were no significant differences in the progression of staining after medicament removal between the simple irrigation method and the EndoActivator Technique.

## 7. Discussion

The primary objective of this research was to compare the extent of discolouration following the removal of three different CAP to a negative control (without any medicament paste). The experimental protocol regulated the amount of simulated sunlight received by the teeth, in comparison to previous studies where the samples were exposed to natural sunlight [9, 10] or ambient laboratory light [31], which would be subject to variations according to the time of day, season, and weather. In the current study, light exposure of 30 minutes per week was designed to not only approximate the possible exposure (total irradiance) of anterior teeth to sunlight but also to standardize the level of light exposure, as this factor has not been well controlled in most earlier work. While the light intensity (power density) used could be higher than what a normal individual may be exposed to (depending on their latitude and outdoor activities), the total irradiance (in Joules) would be realistic, allowing the experiment to accelerate discoloration to a useful end point during the study period of 6 months. The current experimental model demonstrated a strong effect of light exposure in enhancing the staining process, consistent with earlier studies [9]. The findings also reinforce the validity of the current model that untreated roots darken slowly during storage under moist dark conditions at body temperature, an effect seen in earlier work [31].

The results of the current study show an effect of the type of CAP used for 2 or 4 weeks (clinically relevant periods of use) on the progression of staining, even after the pastes have been removed from the root canal. Because the three medicament pastes used in the study contained the same carrier (polyethylene glycol) and included the same corticosteroid component, differences between the effects seen can be attributed to the different antibiotic components. Ledermix paste contains the tetracycline demeclocycline, and this CAP has shown staining of roots in past studies [7, 9, 10, 21, 32]. This makes it suitable as a positive control. There are no previous studies on the medium-term (6 months) effects of either Odontopaste or DoxyPaste, as these are relatively new materials.

The results indicate considerable differences between the two tetracyclines demeclocycline and doxycycline in terms of the severity of discolouration which occurs in roots over time, even when efforts are made to remove remnants of the paste from the root canal system. A recent investigation study revealed that Ledermix is particularly light sensitive, with the material darkening visibly over a period of 45 minutes on the bench under the same light source used in the present study, whereas only a small change was seen in DoxyPaste and no change in Odontopaste under the same irradiation conditions [33]. Of note, the material itself may darken more than the adjacent root, as has been shown in the recent study by Thomson et al. (2012) [31]. The current model uses high intensity light that replicates ground level sunlight however this cannot fully replicate a real world scenario. This model was used to show that despite best methods to remove medicaments, there might be a continued risk of tooth discoloration if light sensitive medicaments are used.

In the study, we tried to imitate clinical conditions in which irrigation may be used to ensure complete removal of medicaments, as well as for ensuring the removal of smear layer and disinfection of the canals. In clinical practice, the use of irrigating systems to remove medicament pastes is not uniform, with some clinicians using EDTA and others NaOCl, and others both in sequence. We therefore used both irrigants alternately in the same pattern as for removal of smear layer and disinfection of the canals, but we recognize that the combination of two agents could contribute to the process of discoloration from the medicament pastes.

The implication of the current findings regarding the use of CAP is that roots treated with Ledermix may continue to discolour over time, despite best efforts being made to remove the material from the canal. This follows on from the propensity of the demeclocycline to chemically interact with the radicular dentine, forming further quinone compounds, which remain despite irrigating away the paste at a later date. It may be appropriate that alternative CAP products to Ledermix be sought for teeth in the aesthetic zone in order to minimise the risks of discolouration.

The concept of trying to limit the contact of Ledermix paste to the middle and lower thirds of the root canal of anterior teeth may be fraught with technical difficulty. Kim et al. observed similar patterns of discolouration in teeth with Ledermix paste in the root canal, with wool placed in the canal orifice, and another group of teeth with Ledermix paste placed in the canals as well as filling the pulp chamber [10]. Several possibilities exist; the tetracycline may have permeated from the paste through the cotton pellet to reach more coronal areas or it may have diffused along the S-shaped dentine tubules of the coronal dentine towards the cementoenamel junction. It is also possible that when placing Ledermix, small amounts of the paste remain on the walls of the pulp chamber despite attempts to remove them.

With regard to the contact time for CAP, the present results indicate that discolouration of tooth structure can occur irrespective of whether the period of application is 2 weeks or 4 weeks. These time periods are consistent with current recommendations, being a minimum of 10 days as being necessary for inflammation to subside [25]. Similarly, two-week duration has been recommended for the placement of intracanal Ledermix [25], as the bacteriostatic efficacy decreases rapidly after the first few days of application [20]. Longer application periods are recommended for periapical or periodontal involvement or if symptoms are not subsiding [25]. The maximal length of duration of Ledermix has been stated to be about two months, though a recent review stated the longest activity for tetracyclines is up to four weeks [34]. The current results indicate that where longer periods of treatment are deemed necessary, the alternative CAP materials of DoxyPaste and Odontopaste would be preferable because of the reduced staining, which would occur.

## 8. Conclusions

The results of the current study indicate that the type of paste influences the pattern of staining which occurs over time, even when considerable efforts have been made to remove remnants of paste using either conventional irrigation or sonically agitated irrigation. There was no additional benefit gained from using the more energetic irrigation protocol. A key point of clinical relevance is that removal of Ledermix did not prevent later discolouration. Because Ledermix paste caused significant darkening to occur after only 2 weeks of contact, the normal use protocols for this material appear to provide sufficient time for the tetracycline in Ledermix paste to incorporate into the radicular dentine. DoxyPaste and Odontopaste were preferable to Ledermix paste in terms of causing less discolouration. The results also reinforce the importance of paste selection and placement for teeth in the aesthetic zone.

## Figures and Tables

**Table 1:** Experimental groups.

Group 1	Negative control with no medicament for 2 weeks (*n* = 10)	
	Negative control with no medicament for 4 weeks (*n* = 10)	

	Part A, (*n* = 60)	Part B, (*n* = 60)
	Simple irrigation removal	EndoActivator removal

Group 2	Odontopaste for 2 weeks	Odontopaste for 2 weeks
	Odontopaste for 4 weeks	Odontopaste for 4 weeks
Group 3	Ledermix for 2 weeks	Ledermix for 2 weeks
	Ledermix for 4 weeks	Ledermix for 4 weeks
Group 4	Doxypaste for 2 weeks	Doxypaste for 2 weeks
	Doxypaste for 4 weeks	Doxypaste for 4 weeks

**Table 2:** Results for normalized luminosity following 2 weeks of paste treatment. Please note lower luminosity values indicate discolouration.

Groups	Baseline	1 month	3 months	6 months
Ledermix-Needle	1.09	0.78	0.78	0.61
	(0.02)	(0.01)	(0.01)	(0.02)
Ledermix-EndoActivator	1.07	0.81	0.79	0.62
	(0.01)	(0.02)	(0.02)	(0.02)
Doxypaste-Needle	1.05	0.96	0.95	0.80
	(0.01)	(0.01)	(0.01)	(0.01)
Doxypaste-EndoActivator	1.07	0.96	0.95	0.82
	(0.01)	(0.01)	(0.01)	(0.01)
Odontopaste-Needle	1.05	1.02	1.02	0.87
	(0.01)	(0.01)	(0.01)	(0.01)
Odontopaste-EndoActivator	1.04	0.99	0.98	0.85
	(0.01)	(0.01)	(0.01)	(0.01)
Control	1.09	1.04	1.02	0.92
	(0.01)	(0.01)	(0.02)	(0.01)

Data show mean and standard deviations.

**Table 3:** Results for normalized luminosity following 4 weeks of paste treatment. Please note lower luminosity values indicate discolouration.

Groups	Baseline	1 month	3 months	6 months
Ledermix-Needle	1.05	0.78	0.72	0.60
	(0.06)	(0.05)	(0.08)	(0.08)
Ledermix-EndoActivator	1.07	0.82	0.75	0.65
	(0.06)	(0.03)	(0.07)	(0.07)
Doxypaste-Needle	1.05	0.93	0.93	0.80
	(0.08)	(0.04)	(0.05)	(0.05)
Doxypaste-EndoActivator	1.08	0.95	0.94	0.83
	(0.06)	(0.03)	(0.03)	(0.03)
Odontopaste-Needle	1.10	1.03	1.02	0.91
	(0.04)	(0.04)	(0.03)	(0.05)
Odontopaste-EndoActivator	1.06	1.00	1.00	0.89
	(0.06)	(0.04)	(0.05)	(0.05)
Control	1.07	1.04	1.02	0.91
	(0.05)	(0.05)	(0.07)	(0.05)

Data show mean and standard deviations.
